# Reduction in undiagnosed HIV infection in the European Union/European Economic Area, 2012 to 2016

**DOI:** 10.2807/1560-7917.ES.2017.22.48.17-00771

**Published:** 2017-11-30

**Authors:** Ard van Sighem, Anastasia Pharris, Chantal Quinten, Teymur Noori, Andrew J Amato-Gauci, the ECDC HIV/AIDS Surveillance and Dublin Declaration Monitoring Networks

**Affiliations:** 1 Stichting HIV Monitoring, Amsterdam, the Netherlands; 2 European Centre for Disease Prevention and Control (ECDC), Stockholm, Sweden; 3 The members of the networks are listed at the end of the article

**Keywords:** HIV, late diagnosis, surveillance, undiagnosed infection, Europe, EU/EEA

## Abstract

It is well-documented that early HIV diagnosis and linkage to care reduces morbidity and mortality as well as HIV transmission. We estimated the median time from HIV infection to diagnosis in the European Union/European Economic Area (EU/EEA) at 2.9 years in 2016, with regional variation. Despite evidence of a decline in the number of people living with undiagnosed HIV in the EU/EEA, many remain undiagnosed, including 33% with more advanced HIV infection (CD4 < 350 cells/mm^3^).

HIV remains an important public health issue affecting the 31 countries of the European Union and European Economic Area (EU/EEA) [1]. In 2015, it was estimated that ca 120,000 people (15% of those living with HIV in the EU/EEA) were living with undiagnosed HIV infection [2]. In order to understand regional variations in (i) HIV incidence, (ii) time to HIV diagnosis, and (iii) the number of people living with undiagnosed HIV, we analysed HIV and AIDS surveillance data from 2003 through 2016.

## Calculation of 2016 estimates

Annually, HIV surveillance data are reported by EU/EEA countries to a database for HIV/AIDS that is coordinated jointly by the European Centre for Disease Prevention and Control (ECDC) and the World Health Organization (WHO) Regional Office for Europe within the European Surveillance System (TESSy) [1].

**Figure 1:**
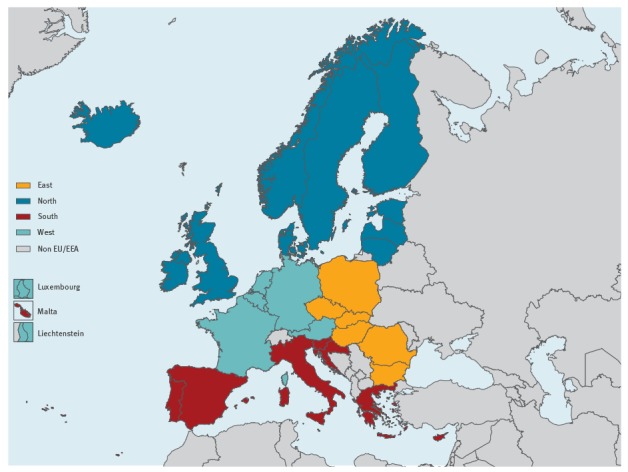
Regions used in the analysis of undiagnosed HIV infections, European Union/European Economic Area, 2016

Countries were grouped into four geographical regions (East, South, West, North) (Figure 1) based on a United Nations definition [3]. For those countries lacking data on CD4 count at diagnosis, the distribution of CD4 count in the region they belonged to was assumed to be representative. When grouping countries, the epidemic characteristics across countries were pooled and a similar probability of diagnosis by CD4 cell count category for all countries within that region was assumed.

EU/EEA: European Union/European Economic Area.

Countries’ grouping based on a United Nations definition [3].

Annual data on HIV diagnoses reported to TESSy for 2003–2016 were adjusted for reporting delay and under-reporting. Data were adjusted for non-national coverage of some countries’ reporting systems (2003–2011 for Italy and 2003–2012 for Spain) and cases (3%) that had been previously reported as diagnosed were excluded. Data were then stratified by the presence of a concurrent AIDS diagnosis and, for people without concurrent AIDS, by CD4 cell count levels at the time of diagnosis i.e. ≥ 500, 350–499, 200–349, < 200 cells/mm^3^ [4]. The ‘incidence method’ in the European Centre for Disease Prevention and Control (ECDC) HIV Modelling Tool version 1.3.0 was used for each region in 2016 to estimate the (i) HIV incidence, (ii) median time from infection to diagnosis, and (iii) number of people living with HIV who were not yet diagnosed [5,6].

## HIV incidence in 2016

In 2016, 29,444 cases of HIV were diagnosed and reported in the EU/EEA, resulting in a notification rate of 5.9 per 100,000 population when adjusted for reporting delay [1]. Rates in 2010–2015 had ranged from 6.5 to 6.7 and in 2016, for the first time in a decade, there was a clear decline in the rate of new HIV diagnoses at EU/EEA level although rates in 11 of 31 EU/EEA countries have continued to increase.

In 2016, the estimated number of new HIV infections (incidence rate) was lower than the number of new diagnoses (notification rate) and was 3.6 per 100,000 population (95% confidence interval (CI): 3.3–4.1) for the EU/EEA overall. This differed substantially between the four regions: the highest estimated incidence rate was in the West (5.2/100,000 population; 95% CI: 4.6–5.9) and South (3.8; 95% CI: 2.8–4.8) and it was lower in the North (2.2; 95% CI: 2.0–3.4) and East (1.7; 95% CI 1.2–2.2).

## Median time from infection to diagnosis

**Figure 2:**
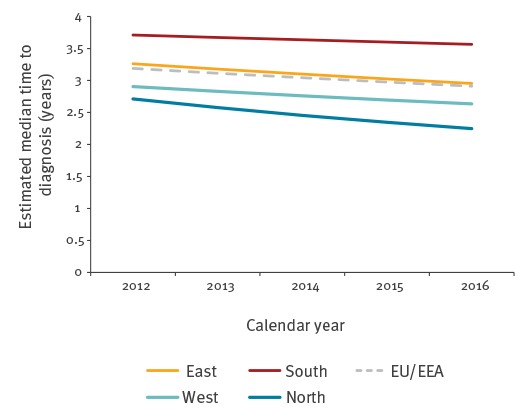
Expected median time from HIV infection to diagnosis by year of infection, European Union/ European Economic Area and regions^a^, 2012-2016

^a^ Countries’ grouping based on a United Nations definition [3].

The median time from infection to diagnosis was estimated to have declined in the EU/EEA overall and in all four regions between 2012 and 2016. In 2016, the EU/EEA median was 2.9 years (interquartile range: 1.4–5.3) with some variation by region: South (3.6 years), East (3.0 years), West (2.6 years) and North (2.2 years) (Figure 2).

**Figure 3:**
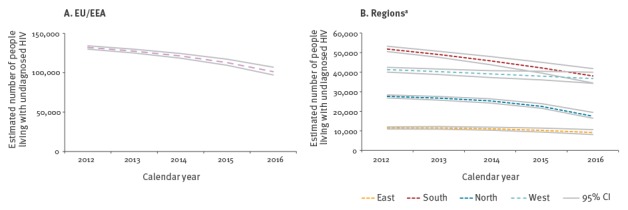
Estimated number of undiagnosed HIV infections, EU/EEA and regions^a^, 2012–2016

The estimated undiagnosed number of people living with HIV in 2016 was highest in the South and West (38,000 and 36,700, respectively), compared with 17,500 in the North and 9,100 in the East (Figure 3). Over the period 2012–2016 the number of people estimated to be living with undiagnosed HIV declined in the EU/EEA overall, from an estimated 132,200 in 2012 to 101,400 in 2016. This decline was observed in all four regions, with a relatively more rapid decline in the South and particularly in recent years in the North. In 2016, this resulted in lower estimated proportions of people living with undiagnosed HIV in the South, North and West (11.0%, 11.3%, and 12.1%, respectively) and a higher proportion in the East (16.8%).

CI: confidence interval; EU/EEA: European Union/ European Economic Area.

^a^ Countries’ grouping based on a United Nations definition [3].

**Figure 4:**
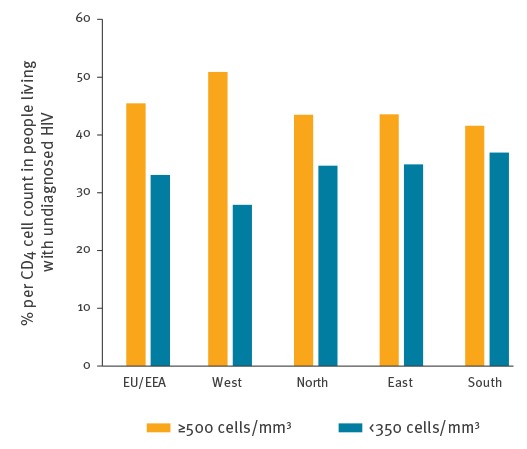
CD4 cell count in people living with undiagnosed HIV, EU/EEA and regions^a^, 2016

We estimated that 45% of those living with undiagnosed HIV in the EU/EEA in 2016 had a CD4 cell count of > 500 cells/mm^3^, which suggests a relatively shorter duration of infection in this group (Figure 4). Conversely, 33% had an estimated CD4 cell count of < 350 cells/mm^3^, indicating a longer duration of HIV infection. Recency of infection varied by region, with the highest proportion of more recent infections observed in the West (51%) and the highest proportion of infections of longer duration in the South (37%).

EU/EEA: European Union/ European Economic Area.

^a^ Countries’ grouping based on a United Nations definition [3].

## Discussion and conclusions

Our analysis provides evidence of a decline in the number of people living with undiagnosed HIV during the last 5 years in the EU/EEA. This decrease was more evident in the South and North regions. This may be associated with observed national-level declines in reported HIV notifications and newly acquired infections, evidence of more frequent testing and earlier diagnosis in some countries [7-10].

With a notification rate of 5.9 per 100,000 population and an estimated incidence of 3.6 per 100,000, it appears that currently more individuals in the EU/EEA are being diagnosed than are newly infected with HIV. Between 2012 and 2016, the yearly diagnosed fraction (yearly number of new diagnoses/(yearly number of new diagnoses + estimated number of undiagnosed people living with HIV)) increased from 20% to 23% [11]. This suggests that testing activities are ‘gaining ground’ on the hidden epidemic. If our estimates are accurate, if incidence does not increase unexpectedly, and if testing patterns remain stable or increase, this should result in further reductions in the number of undiagnosed people living with HIV and indicate progress towards achieving the global 2020 target of 90% of people living with HIV in the EU/EEA being diagnosed [12]. Currently there is heterogeneity across regions with regard to this target, with faster proportional declines in the number of people living with undiagnosed HIV in the South and North regions. The highest proportions of people living with undiagnosed HIV are in the countries in the East region of the EU/EEA.

Despite the welcome and positive trend in reduced numbers of undiagnosed infections, we estimated that it takes a median of 2.9 years from infection to diagnosis, and even more than 3 years in some regions of the EU/EEA. Shorter time to diagnosis is well-correlated with a higher CD4 cell count at diagnosis in the various regions; while the reverse is also true. It is well documented that early HIV diagnosis and rapid linkage to antiretroviral treatment for HIV results in lower HIV-related morbidity and mortality for individuals infected [13], and in reduced HIV transmission at population-level [14]. Diversifying and streamlining HIV testing programmes may improve access and uptake. Effective ways of doing this include augmenting routine testing for health conditions associated with HIV (‘indicator condition-guided testing’), increasing HIV testing during screening for other sexually transmitted infections, and continuing to expand community-based testing, self-testing/home-sampling, and partner notification [15-17].

Our analysis is subject to several important limitations. First, it is based on surveillance data and, while the quality of EU/EEA surveillance data are generally good and continue to improve year-on-year, some countries do not report CD4 cell count at diagnosis. Our assumption that the distribution of CD4 cell count was the same in these countries as for others in the same region could have resulted in less robust estimates by region. Second, the effect of migration on HIV incidence and the undiagnosed number is complex and this was not accounted for in this analysis. Third, there is substantial variation in epidemic patterns within the four regions and overall results for a region cannot be directly applied to the individual countries within it with confidence. Often estimates for the region were heavily influenced by the countries with larger population within the region. Still, our exercise points to the diversity of HIV incidence and in the undiagnosed population across Europe and underlines the need for increased availability of robust national estimates to guide and evaluate testing and prevention practice.

This analysis provides evidence that, although patterns across Europe are diverse, there appears to be a positive trend towards lower numbers of people living with undiagnosed HIV over time. Still, differences across the EU/EEA persist and these trends are not seen to the same extent in all the four regions analysed. We found that a substantial number of people are living with undiagnosed HIV in the EU/EEA, that 33% of these have more advanced HIV infection, and that the median time from infection to diagnosis is still nearly 3 years. Together this indicates that additional efforts are needed to increase the availability and uptake of HIV testing and rapid linkage to HIV treatment in countries in the EU/EEA.

## Notes

**The ECDC HIV/AIDS Surveillance and Dublin Declaration Monitoring Networks:** ECDC HIV/AIDS Surveillance and Dublin Declaration Monitoring Networks include: Austria: Daniela Schmid, Irene Rueckerl, Robert Zangerle; Belgium: Andre Sasse, Dominique Van Beckhoven, Frédéric Denauw; Bulgaria: Tonka Varleva, Vyara Georgieva; Croatia: Tatjana Nemeth Blazic, Jasmina Pavlic, Josip Begovac; Cyprus: Maria Koliou, Linos Hadjihannas, Anna Demetriou; Czech Republic: Marek Maly, Vratislav Němeček, Veronika Šikolová; Denmark: Susan Cowan, Jan Fouchard; Estonia: Kristi Rüütel, Anna-Liisa Pääsukene; Finland: Kirsi Liitsola, Mika Salminen, Henrikki Brummer- Korvenkontio; France: Françoise Cazein, Josiane Pillonel, Florence Lot, Jean- Christophe Comboroure; Germany: Barbara Gunsenheimer-Bartmeyer, Matthias an der Heiden, Gesa Kupfer, Ulrich Marcus; Greece: Vasilios Raftopoulos, Stavros Patrinos, Vasileia Konte; Hungary: Maria Dudas, Katalin Szalay; Iceland: Haraldur Briem, Gudrun Sigmundsdottir; Ireland: Derval Igoe, Kate O’Donnell, Caroline Hurley, Fiona Lyons; Italy: Barbara Suligoi, Anna Caraglia; Latvia: Šarlote Konova; Liechtenstein: Sabine Erne; Lithuania: Irma Čaplinskienė; Luxembourg: Aurelie Fischer, Patrick Hoffmann; Malta: Jackie Maistre Melillo, Tanya Melillo; Netherlands: Eline Op de Coul, Silke David; Norway: Hans Blystad, Arild Johan Myrberg; Poland: Magdalena Rosinska, Iwona Wawer; Portugal: Isabel Aldir, Helena Cortes Martins, Teresa Melo; Romania: Mariana Mardarescu; Slovakia: Peter Truska, Jan Mikas; Slovenia: Irena Klavs; Spain: Asuncion Diaz, Olivier Nuñez, Olivia Castillo; Sweden: Maria Axelsson, Anders Sönnerborg; United Kingdom: Peter Kirwan, Cuong Chau, Sandra Okala, Alison Brown, Valerie Delpech.
